# 3D Printing Fiber Electrodes for an All‐Fiber Integrated Electronic Device via Hybridization of an Asymmetric Supercapacitor and a Temperature Sensor

**DOI:** 10.1002/advs.201801114

**Published:** 2018-09-25

**Authors:** Jingxin Zhao, Yan Zhang, Yinan Huang, Jixun Xie, Xiaoxin Zhao, Chaowei Li, Jingyi Qu, Qichong Zhang, Juan Sun, Bing He, Qiulong Li, Conghua Lu, Xinhua Xu, Weibang Lu, Liqiang Li, Yagang Yao

**Affiliations:** ^1^ School of Materials Science and Engineering Tianjin University Tianjin 300072 P. R. China; ^2^ Division of Advanced Nanomaterials Key Laboratory of Nanodevices and Applications CAS Center for Excellence in Nanoscience Suzhou Institute of Nano‐tech and Nano‐bionics Chinese Academy of Sciences Suzhou 215123 P. R. China; ^3^ Division of Nanomaterials Suzhou Institute of Nano‐Tech and Nano‐Bionics Nanchang, Chinese Academy of Sciences Nanchang 330200 China

**Keywords:** 3D printing, fiber‐shaped asymmetric supercapacitors, fiber‐shaped integrated electronic devices, fiber‐shaped temperature sensors, V_2_O_5_/ single‐walled carbon nanotubes (SWCNTs) fibers

## Abstract

Wearable fiber‐shaped electronic devices have drawn abundant attention in scientific research fields, and tremendous efforts are dedicated to the development of various fiber‐shaped devices that possess sufficient flexibility. However, most studies suffer from persistent limitations in fabrication cost, efficiency, the preparation procedure, and scalability that impede their practical application in flexible and wearable fields. In this study, a simple, low‐cost 3D printing method capable of high manufacturing efficiency, scalability, and complexity capability to fabricate a fiber‐shaped integrated device that combines printed fiber‐shaped temperature sensors (FTSs) with printed fiber‐shaped asymmetric supercapacitors (FASCs) is developed. The FASCs device can provide stable output power to FTSs. Moreover, the temperature responsivity of the integrated device is 1.95% °C^−1^.

## 1 Introduction

With the rapid development of flexible and portable electronics, wearable energy storage devices have become an important component of modern electronics.^1^, ^2^, ^3^, ^4^, ^5^ As one of the most promising power sources, electrochemical supercapacitors have been widely used in wearable electronics due to their high power density, excellent reversibility, large capacity, low cost, and long lifecycle.^6^, ^7^, ^8^, ^9^, ^10^ However, their low energy density has limited their further development in many fields. The recent emergence of fiber‐shaped asymmetric supercapacitors (FASCs) has considerably resolved the problems with the low energy density of electrochemical supercapacitor devices.^11^, ^12^, ^13^

The active materials play a key role in an electrochemical supercapacitive system, and many efforts have been dedicated in recent years to the development of various electrochemically active substances and smart designs in FASC devices to enable wearable and flexible electronic devices. For example, Peng and co‐workers successfully fabricated various wearable fiber‐shaped supercapacitors by combining aligned multiple‐walled carbon nanotube fibers with electrochemical active materials consisting of ordered mesoporous carbon, MoS_2_, and polyaniline (PANI), which all exhibit excellent electrochemical performance.^14^, ^15^, ^16^ Chou and co‐workers demonstrated the construction of a stretchable wire‐shaped asymmetric supercapacitor based on pristine and MnO_2_‐coated carbon nanotube fibers, and the specific capacitance of the as‐fabricated stretchable device was almost unchanged with a cyclic tensile strain of up to 100%.^9^ Qu and co‐workers constructed all‐graphene, core–sheath, all‐solid‐state, stretchable fibriform supercapacitors and wearable electronic textiles via a facile one‐step dimensionally confined hydrothermal strategy from aqueous suspensions; this strategy can be managed to produce highly compressible and stretchable spring supercapacitors and can also be woven into a textile for wearable electronics.^17^ Gao and co‐workers developed a coaxial wet‐spinning assembly approach to continuously spin polyelectrolyte‐wrapped graphene/carbon nanotube core–sheath fibers that are used directly as safe electrodes in the assembly of two‐ply yarn supercapacitors.^18^ However, these strategies suffered from the persistence of abundant limitations in fabrication cost, efficiency, preparation procedure, and scalability that have impeded their practical application. As a result, the evolution of 1D FASCs via a simple, low‐cost method with high manufacturing efficiency, scalability, and complexity capability remains a challenge.

3D printing is one of the most advanced additive manufacturing technologies, and it has attracted considerable attention from industry and academia for the development of advanced manufacturing materials due to its high manufacturing efficiency, scalability, low cost, and complexity capability.^19^, ^20^, ^21^, ^22^ This technique has already been developed for a wide range of applications, such as energy, engineered composites, microfluidics, biotechnology, and electronic devices.^23^, ^24^, ^25^ Very recently, interesting studies have emerged. Chen and co‐workers formed 3D printed microsupercapacitors based on graphene and showed excellent electrochemical performance.^26^ However, the larger planar structure limits the development of wearable energy storage devices. Therefore, it is necessary to construct a 3D‐printed fiber‐shaped wearable energy storage device.

Human beings have always attempted to purse more comfortable and fascinating lives. Temperature sensors have emerged at the right moment to detect temperature variations and prevent the advent of disease.^27^, ^28^ Wearable temperature sensors capable of real‐time monitoring of human health‐related parameters can offer new approaches to manage the health status and performance of individuals to enable many emerging applications, such as e‐skin, smart watches, robot sensors, human–machine interfaces, health care, human activity monitoring, and environmental temperature measurement.^29^, ^30^, ^31^, ^32^ However, wearable temperature sensors have been mainly based on planar structure, and fiber‐shaped structures have rarely been reported. In addition, reduced graphene oxide (rGO) serves as a highly sensitive material for the detection of temperature changes due to its adjustable bandgap and activation energy.^33^ Thus, the construction of flexible, wearable, highly sensitive, and lightweight FTSs based on rGO is a top priority. The integration of energy harvest, storage, and conversion into a single configuration has attracted abundant attention in recent years.^34^, ^35^, ^36^, ^37^, ^38^ Our target in adopting this strategy is to integrate FTSs with FASCs to fabricate a device that can provide stable output power to FTSs. As a conceptual exhibition, a high‐performance, 3D printed, fiber‐shaped integrated electronic device was fabricated. In this energy storage and conversion configuration, a FASCs device with a maximum operating voltage of 1.6 V was assembled by twisting a single‐walled carbon nanotube (SWCNT)/V_2_O_5_ fiber cathode and an SWCNT/VN fiber anode. The as‐fabricated FASCs device can drive the FTSs, and the fiber‐shaped integrating electronic device shows great sensitivity in the detection of ambient temperature, with a temperature responsivity of 1.95% °C^−1^.

## 2 Results and Discussion

**Figure 1:**
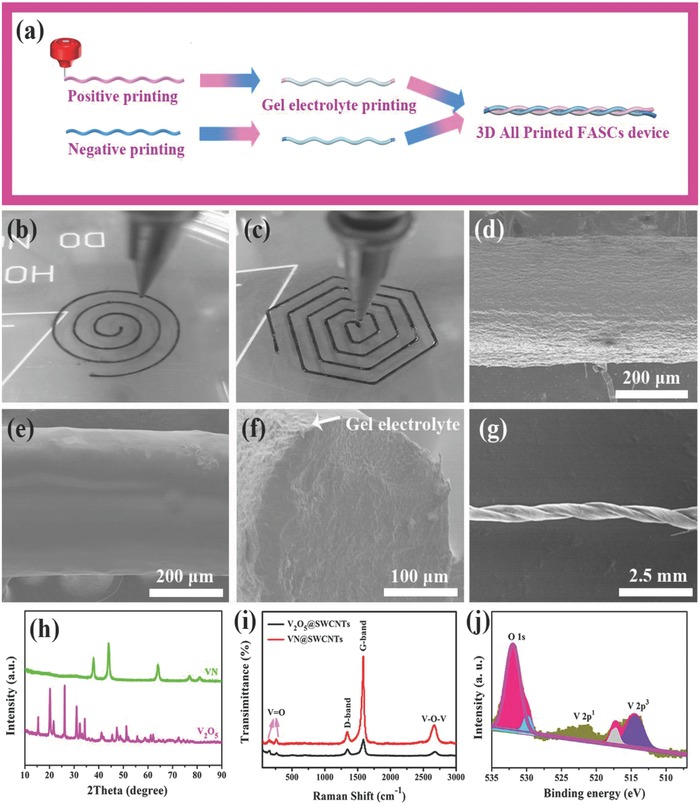
Fabrication process and structural characterization of the FASCs. a) Schematic of the fabrication process of the 3D‐printed FASC device. b,c) Optical images of the wet fiber during the printing process. d) SEM image of the V_2_O_5_/SWCNT fiber. e) SEM image of gel electrolyte coated on the fiber. f) Cross‐sectional SEM image of gel electrolyte coating on the fiber. g) SEM image of the assembled FASC device. h) XRD patterns of the as‐prepared V_2_O_5_ and VN samples. i) Raman spectra of as‐prepared V_2_O_5_/SWCNTs and VN/SWCNT fibers. j) XPS spectra of the as‐prepared V_2_O_5_/SWCNTs.

A series of procedures were executed to fabricate the 3D‐printed FASC device. First, the V_2_O_5_ and VN samples (fabricated by electrospinning method) and the structure characterization (field emission scanning electron microscopic (FESEM) and transmission electron microscopic (TEM) images) depicted in Figure S1 (Supporting Information), were added to the solution with an SWCNT conductive additive to obtain the ideal preprinted positive and negative inks (the details in the Experimental Section), respectively. Second, electrode materials of V_2_O_5_ and VN samples were formed into fibers via 3D printing technology. Third, the polyvinyl alcohol (PVA)/KOH gel electrolyte was printed in as‐printed positive and negative fibers, respectively. Finally, the two fibers were twisted, and the fiber‐shaped FASC device was successfully fabricated. The schematic diagram for 3D printing and the device's assembly process is depicted in **Figure** **1**a. Unlike traditional solution spinning, CNT‐based electrode fibers could be fabricated into a variety of patterns. As shown in Figure 1b,c, positive and negative electrode fibers were molded into an Archimedean spiral and a six‐angle spiral, respectively. This spinning used a typical embedded 3D printing process, and the embedded 3D printer is self‐made. Both inks were extruded below the liquid surface of the coagulation bath. When the inks were ejected into the coagulation bath, the PVA in the coagulation bath replaced the surfactant in the electrode inks. Therefore, a few minutes were needed for the fiber to completely solidify. In the extrusion process, SWCNTs and active materials were oriented along the extrusion direction, thus increasing the fibers' mechanical strength. Here, we denoted our 3D‐printed positive electrode and negative electrode as V_2_O_5_/SWCNTs and VN/SWCNTs, respectively. The morphology and microstructure of the as‐printed V_2_O_5_/SWCNTs fibers were characterized by FESEM, as shown in Figure 1d–f. Figure 1d shows a SEM image of a single V_2_O_5_ positive fiber with a diameter of ≈250 µm. A SEM image of the as‐printed gel electrolyte on fiber is shown in Figure 1e, and the cross‐sectional SEM image in Figure 1f reveals that the V_2_O_5_ samples are uniformly and tightly wrapped by the gel electrolyte and that the gel electrolyte has a porous structure layer with a thickness of 8–16 µm, which can result in the electrode fiber making intimate contact with the electrolyte and facilitating electrolyte penetration. Meantime, the microstructure and morphology of VN/SWCNT‐negative fiber and pristine SWCNT fiber are observed in Figure S2 (Supporting Information). Figure 1g shows the assembled device's twisted fiber structure. Additionally, the mechanical tensile test results show the excellent flexibility and mechanical strength of the printed fibers (Figure S3, Supporting Information). The crystal structure and phase structure of as‐prepared V_2_O_5_ and VN fibers are further characterized by X‐ray diffraction (XRD) (Figure 1h), which reveals the V_2_O_5_ and VN to be orthorhombic and cubic crystal structures, respectively. Figure 1i shows the Raman spectra of V_2_O_5_ and VN samples, exhibiting typical peaks VO*_x_*, VO*_x_* coated on VN and the characteristic peaks of carbon (with the G‐band and the D‐band at around 1346 and 1588 cm^−1^, respectively).^11^ X‐ray photoelectron spectroscopy (XPS) was conducted to investigate the surface element of as‐prepared V_2_O_5_ fiber and VN fiber. Figure 1j shows three peaks at 514.0, 521.2, and 531.8 eV corresponding to the binding energy of V 2p^3^, V 2p^1^, and O 1s electrons in the range of 510–535 eV, respectively.^11^ The main XPS peaks at 284.8 and 399 eV correspond to the C 1s level of carbon and the N 1s level of VN, respectively (Figure S4a,b, Supporting Information).^13^

**Figure 2:**
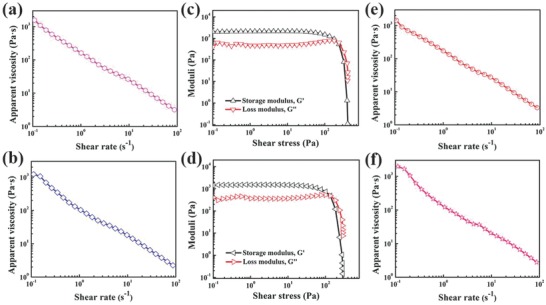
Rheological properties of V_2_O_5_/SWCNTs and VN/SWCNT slurry inks. a,b) Apparent viscosity as a function of shear rate for V_2_O_5_/SWCNTs and VN/SWCNT inks, respectively. c,d) Storage modulus, *G*′, and loss modulus, *G*″, as a function of shear stress for V_2_O_5_/SWCNT inks and VN/SWCNT inks, respectively. e,f) Apparent viscosity as a function of shear rate for V_2_O_5_/SWCNTs and VN/SWCNT inks, respectively, after four weeks of storage.

For the fabrication of ideal 3D printing inks, the rheological properties and stability should be given priority. **Figure** **2**a,b shows the apparent viscosity as a function of the shear rate for positive and negative inks, respectively. Each ink exhibits typical shear‐thinning behavior, which is very favorable for the extrusion processing of high‐viscosity inks.^39^ This shear‐thinning behavior originates mainly from the orientation of the SWCNTs and pseudocapacitance material during extrusion (SEM images of V_2_O_5_/SWCNTs and VN/SWCNT fibers are exhibited in Figure S5, Supporting Information). The storage modulus (*G*′) and loss modulus (*G*″) of the positive and negative inks as a function of shear stress are shown in Figure 2c,d. For both electrode inks, the storage modulus is greater than the loss modulus under 10^3^ Pa, which reflects the solid‐like response and is crucial for solidification of fiber. As the shear stress increases, the elastic modulus decreases more than the loss modulus, and the inks exhibit liquid‐like behavior. In this region, inks mainly exhibit viscous deformation, which is conducive to the extrusion 3D printing process. In addition, our electrode inks also show excellent time stability. As shown in Figure 2e,f, the viscosity of each ink is nearly constant after four weeks of static storage, which indicates the stability of the rheological properties under long time storage. Compared with printable inks, the pure slurry demonstrates a considerable plateau value for its *G*′ and *G*″ (Figure S6, Supporting Information), which indicates that the addition of active substances has no effect on the rheological properties. In addition, the electrolyte ink also embraces the good rheological property (Figure S7, Supporting Information).

To survey the electrochemical properties of the as‐printed V_2_O_5_ fiber positive electrode material and the VN fiber negative electrode material, measurements via cyclic voltammetry (CV), galvanostatic charge/discharge (GCD), and electrochemical impedance spectroscopy (EIS) were weighed in a three‐electrode configuration with a 1 m KOH electrolyte. Figure S8a (Supporting Information) shows the CV image of the V_2_O_5_/SWCNT fiber positive electrode in comparison with the pure SWCNT fiber (SEM image in Figure S9, Supporting Information) electrode obtained at a scan rate of 25 mV s^−1^ (Supporting Information). The signal from the pure SWCNT fiber electrode was negligible compared with the V_2_O_5_/SWCNT fiber positive electrode. Figure S8b (Supporting Information) displays the CV curves of the as‐fabricated V_2_O_5_/SWCNT fiber positive electrode at various scan rates (5, 20, 50, 100, 200, and 500 mV s^−1^) between 0 and 0.6 V (vs SCE), which exhibits typical faradaic pseudocapacitive behavior. For the V_2_O_5_/SWCNT fiber positive electrode, the maximum areal capacitance value at 0.5 mA cm^−2^ is 587.93 mF cm^−2^, and a specific capacitance of 440 mF cm^−2^ is obtained at the current density of 6.4 mA cm^−2^, which indicates good rate capability (Figure S8c, Supporting Information). The as‐obtained positive electrode has a smaller intrinsic resistance (*R*_b_) value of ≈61.78 Ω (Figure S8d, Supporting Information). Meantime, the electrochemical performance of the as‐printed VN/SWCNT fiber negative electrode was also accurately assessed (Figure S10, Supporting Information), and the corresponding details are described in the Supporting Information.

**Figure 3:**
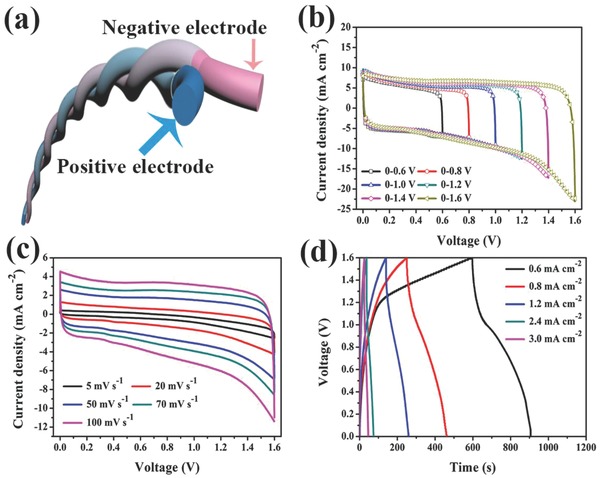
Assembled 3D‐printed FASC device. a) Schematic of the assembled structure of the FASCs. b) CV of the assembled device measured for different voltage windows. c) CV curves of the assembled FASCs at various scan rates. d) GCD curves of the assembled FASCs at different current densities.

A flexible and wearable FASC device was assembled by twisting together the 3D‐printed V_2_O_5_/SWCNTs and the VN/SWCNT fiber electrode (**Figure** **3**a). The occlusive area of the CV curves (Figure 3b) of the as‐assembled FASC device increases with the range of voltage windows, increasing from 0.6 to 1.6 V at a scan rate of 25 mV s^−1^ in a two‐electrode system. A quasirectangular shape is observed in the CV curves (Figure 3b) of the assembled FASC device, which shows ideal capacitive behavior from the synergistic effects of the two fiber‐shaped electrodes.^11^ The CV, GCD, and electrochemical impedance spectroscopic images were also surveyed to further investigate the electrochemical performance of the as‐fabricated device. Figure 3c shows that the CV curves of the obtained FASC device retain their quasirectangular shape when the sweep rate increases from 5 to 100 mV s^−1^ for the operating voltage windows between 0 and 1.6 V, which indicates that the obtained FASC device possesses excellent electrochemical reversibility.^12^ The specific capacitance of the as‐obtained FASC device was calculated by GCD curves (Figure 3d), which reveal that the highest specific capacitance is 116.19 mF cm^−2^ at a current density of 0.6 mA cm^−2^. The equivalent series resistance of the FASC device was examined, and a value of ≈20.76 Ω was obtained from the Nyquist plot, as shown in Figure S11 (Supporting Information).

**Figure 4:**
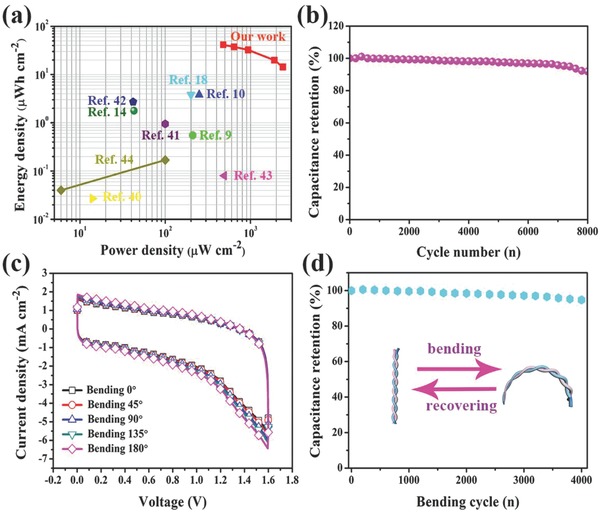
Assembled 3D‐printed FASC device. a) Ragone plot (power density vs energy density) of FASC device. b) Cyclic stability. Flexible and mechanical tests of assembled FASC device. c) CV curves of FASCs with different bending angles. d) Capacitance retention after 4000 cycles of bending.

In the Ragone plots (**Figure** **4**a) of the assembled FASC device, the highest areal energy density (*E*_A_) of 41.28 µWh cm^−2^ was obtained at an areal power density (*P*_A_) of 480 µW cm^−2^, which indicates a good synergistic effect of the as‐printed V_2_O_5_/SWCNT fiber positive electrode and VN/SWCNT fiber negative electrode. The *E*_A_ and *P*_A_ values of our FASC device are higher than the great majority of fiber‐shaped supercapacitors, such as wire‐shaped asymmetric supercapacitors based on pristine, MnO_2_‐coated CNTFs (*E*_A_ = 0.55 µWh cm^−2^ and *P*_A_ = 210 µW cm^−2^);^9^ wet‐spinning rGO/CNTFs (*E*_A_ = 3.84 µWh cm^−2^ and *P*_A_ = 200 µW cm^−2^);^18^ ZnO nanowire‐coated polymer fiber‐based supercapacitors (*E*_A_ = 0.027 µWh cm^−2^ and *P*_A_ = 14 µW cm^−2^);^40^ yarn supercapacitors based on CNTs and ordered mesoporous carbon (*E*_A_ = 1.77 µWh cm^−2^ and *P*_A_ = 43 µW cm^−2^);^14^ polyaniline coated on stainless steel wire‐based supercapacitors (*E*_A_ = 0.95 µWh cm^−2^ and *P*_A_ = 100 µW cm^−2^);^41^ Ni wire/carbon ink‐based supercapacitors (*E*_A_ = 2.7 µWh cm^−2^ and *P*_A_ = 42 µW cm^−2^);^42^ CNTs/rGO composite fiber‐based supercapacitors (*E*_A_ = 3.8 µWh cm^−2^ and *P*_A_ = 250 µW cm^−2^);^10^ CNT fiber‐based stretchable wire‐shaped supercapacitors (*E*_A_ = 0.08 µWh cm^−2^ and *P*_A_ = 493 µW cm^−2^);^43^ and all‐graphene core–sheath microfiber‐based supercapacitors (*E*_A_ = 0.04–0.17 µWh cm^−2^ and *P*_A_ = 6–100 µW cm^−2^).^44^ In addition, the capacitance retention rate of the fully 3D‐printed FASC device can reach 91.9% after 8000 cycles at a current density of 0.8 mA cm^−2^ (Figure 4b). The flexibility and mechanical stability are important factors for the FASC device. From Figure 4c, it can clearly be seen that the shapes of the CV curves do not change significantly with bending angels of 0°, 45°, 90°, 135°, and 180° at a scan rate of 25 mV s^−1^, indicating the splendid flexibility and mechanical stability of the fully printed FASC device. Moreover, 94.7% of capacitance reservation rate is obtained after repetitive bending for 4000 bending cycles at a bending angle of 45° (Figure 4d), and the electrical properties of V_2_O_5_/SWCNTs and VN/SWCNTs is also investigated under bending deformation (Figure S12, Supporting Information).

**Figure 5:**
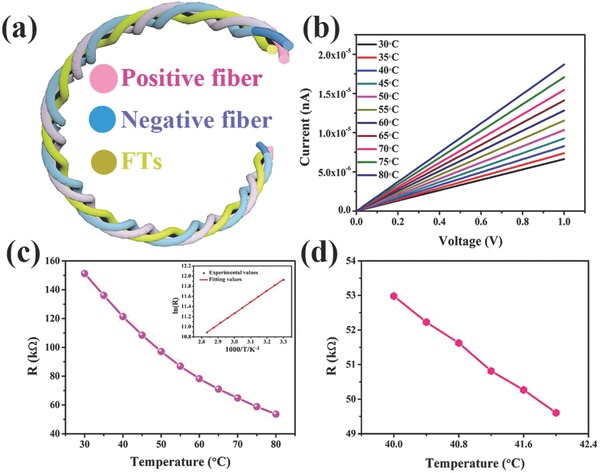
Integrated configuration. a) Schematic diagram of FASC device/FTS‐integrated configuration. b) *I–V* curves of the integrated device between 30 and 80 °C (temperature steps set as 5 °C for clarity). c) Relationship between resistance of the integrated electronic device and temperature. (Inset) Fitting curves for ln(*R*) versus 1/*T*. d) Response resistance as a function of temperature (temperature steps set as 0.4 °C for clarity).

A 3D‐printed, flexible, and wearable fiber‐shaped integrated electronic device was constituted with a FASC device and an FTS based on rGO fiber (Figure S13, Supporting Information, shows the photograph of FASCs/FTSs integrated configuration). **Figure** **5**a shows a schematic diagram of the FASCs/FTS integrated configuration. The structural characterization and rheological properties of as‐prepared graphene fiber are exhibited in Figure S14 (Supporting Information). The FASC device can offer a voltage of 1.6 V to supply the power for the FTSs. Figure 5b reveals the *I–V* (current–voltage) curves of the integrated device when the temperature increased from 30 to 80 °C (plotting step is 5 °C). The current of the integrated configuration increased from 6.56 µA at 30 °C to 18.68 µA at 80 °C at a fixed voltage of 1 V, which indicates that the low operating voltage of the device can be provided by FASCs. To investigate the ability of the integrated electronic device to monitor human temperature with high accuracy, the thermal response of our wearable fiber‐shaped integrated device was examined. Figure S15 (Supporting Information) displays the current increases in a linear manner with the voltage, which demonstrates that the conductive behavior of the integrated configuration is ohmic contact. Figure 5c indicates that the resistance of the integrated electronic device decreases as the temperature increases. The resistance of the integrated configuration decreases from 151.17 kΩ at 30 °C to 53.6 kΩ at 80 °C. The responsivity of the temperature sensor is important, and the responsivity of the integrated device is defined as ((*R* −*R*_0_)/*R*_0_) × 100%, where *R*_0_ and *R* are the resistances at 30 °C and the set temperature up to 80 °C, respectively. The temperature sensitivity of the integrated device was 1.95% °C^−1^ extracted from Figure 5c, the value of which was larger than those of the reported CNTs (−7 × 10^−4^ °C^−1^) and the standard commercial platinum temperature sensors (39.2 × 10^−4^ °C^−1^), a linear relationship between ln(*R*) and 1000/*T* (inset of Figure 5c) shows a good fit with Arrhenius equation, suggesting that the temperature response of rGO fiber is primarily due to thermal activation leading to the generation of electron and hole pairs, similar to intrinsic semiconducting behaviors that comply with hopping transport. To detect the environmental thermal irradiation, the integrated device was irradiated with an infrared light (see Figure S16, Supporting Information, inset). As shown in Figure S16 (Supporting Information), the output sensing signal increases to the maximum continuously as the infrared irradiation time is prolonged, and the output sensing signal returns to its initial value when the infrared irradiation stops, thus demonstrating a precise and continuous thermal characterization ability. In addition, it is quite obvious that our integrated device can respond and recover very quickly within a few seconds, which is beneficial for the wearable temperature sensing device (Figure S17, Supporting Information). The typical response resistance as a function of temperature, measured over a small temperature range of 40–42 °C with intervals of 0.4 °C, as shown in Figure 5d, discloses the high‐temperature resolution and precise measurement of our integrated device. Furthermore, the repeatability of the integrated configuration is also exhibited in Figure S18 (Supporting Information). Figure S19 (Supporting Information) shows the response resistance of the FTSs as a function of humidity, and the slight humidity changes cause the tiny resistance changes, which can be ignored relative to the temperature response.

## 3 Conclusion

In summary, we first used advanced 3D printing technology to rapidly construct a 3D‐printed fiber‐shaped integrated electronic device that integrates the printed FTSs with the printed FASC, and the FASC device can provide stable output power to the FTSs. In the FASC device, the V_2_O_5_/SWCNT fiber positive electrode and VN/SWCNT fiber negative electrode were twisted, and the FASC device exhibits a high specific capacitance of 116.19 mF cm^−2^ at a current of 0.6 mA cm^−2^ and excellent mechanical flexibility. Meantime, the temperature sensitivity of the integrated configuration is 1.95% °C^−1^. Therefore, the rapid, accurate, scalable, and low‐cost 3D printing technique will open new opportunities for flexible, wearable, fiber‐shaped, integrated electronic devices.

## 4 Experimental Section

*Preparation of V_2_O_5_ and VN Hollow Fibers*: According to a previous report,^45^ C_2_H_2_O_4_·2H_2_O (2.72 g) and NH_4_VO_3_ (1.25 g) were added to a mixture of 10 mL of C_2_H_5_OH and 10 mL of water. The solution was stirred for clarification, and then 1.1 g of polyvinylpyrrolidone (PVP) was added into the above solution to increase viscosity. Subsequently, the mixture was magnetically stirred for several hours at room temperature to form a homogeneous precursor solution. In a typical electrospinning process, the as‐obtained spinnable sol was loaded into a plastic syringe equipped with a 7# stainless steel needle. A high voltage of 15 kV was supplied by a direct‐current power supply. The distance between the tip of the needle and collector was 15 cm. For the following thermolysis process, as‐spun nanofibers were placed in a muffle furnace and calcined at 400 °C for 15 min with a heating rate of 0.5 °C min^−1^ to remove PVP and obtain V_2_O_5_ hollow fibers. In order to obtain VN hollow fibers, V_2_O_5_ hollow fibers were heated up to 600 °C for 1 h with a heating rate of 2.0 °C min^−1^ and cooled the furnace naturally under NH_3_ flow overnight.

*The Preparation of Inks and Coagulation Bath*: For the CNTs‐based electrode fiber, coagulation bath used here is 5% PVA aqueous solution. 5 g PVA powder was added into 95 mL deionized water and then stirred for 3 h at 80 °C. The SWCNTs dispersion was obtained by dispersing SWCNT (1000 mg) and PVA (200 mg) in N‐methyl‐2‐pyrrolidone (NMP) (10 mL) and grinding for 1 h under a fume hood. Then pseudo‐capacitance materials (VN or V_2_O_5_ powder) (200 mg) were added to the resultant paste. Finally, the electrode inks were concentrated to 200 mg mL^−1^. For the GO fiber, coagulation bath was prepared by mix 5 g CaCl_2_, 75 mL H_2_O, and 25 mL ethanol. GO was obtained by a modified Hummers' method.^41^ The concentration of GO ink is 20 mg mL^−1^.

*3D Printing Fiber*: A self‐built multiaxis micropositioning system (Prusa i3) was utilized for printing with the nozzle modified into screw extrusion. SWCNTs‐based electrode and GO inks were all housed in 1 mL syringe. The nozzle of electrode inks is 410 µm and the nozzle of GO ink is 500 µm. For each inks, the nozzle was immersed in coagulation bath with a typical move speed of 8 mm s^−1^. After printing, the fiber was immersed in coagulation bath for 5 min to remove solvent followed by washing with ethanol and water, and dried for 24 h at room temperature. The rGO fibers were prepared by chemical reduction of the obtained GO fibers in aqueous HI at 80 °C for 8 h, followed by washing with ethanol and vacuum drying for 12 h.

*Characterization*: XRD measurements were performed using an X‐ray diffractometer using Cu Kα radiation (D8 Advance PANalytical X'Pert Pro) from 10° to 80°. The morphology and microstructures of the samples were observed by a FESEM (S‐4800). The Raman spectra of the V_2_O_5_/SWCNTs and VN/SWCNTs fibers were recorded using a micro‐Raman spectroscope (LABRAM HR, excitation wavelength of 532 nm). XPS was performed on an ESCALab MKII X‐ray photoelectron spectrometer with nonmonochromatized MgKα X‐ray as the excitation source. The binding energies in the XPS analysis were corrected by referencing C 1s to 284.6 eV. Atomic force microscopy (AFM) images were taken in the tapping mode by carrying out on a NSK SPI3800.

*Assembly of Flexible FASCs Device*: A solid gel electrolyte was prepared by slowly adding 8 g PVA to 100 mL of deionized water, followed by heating at 95 °C and vigorous magnetic stirring until the solution became clear. After the PVA gel was cooled to room temperature, a saturated aqueous solution containing 11.2 g of KOH was added with strong magnetic stirring. Thus, the PVA‐KOH gel electrolyte was obtained. For uniformity, the gel electrolyte was printed on V_2_O_5_/SWCNTs positive electrode and VN/SWCNTs negative electrode fiber, respectively. FASCs device was assembled by twisting the V_2_O_5_/SWCNTs fiber@gel electrolyte and VN/SWCNTs@gel electrolyte.

*Electrochemical Measurements*: For all electrochemical measurements (CV, GCD, and EIS), each electrode material was assessed using an electrochemical working station (CHI760E, Shanghai, China) in a three‐electrode system in 1 m KOH aqueous electrolyte at room temperature. The average specific capacitance values of the electrodes and the asymmetric supercapacitor devices were calculated from GCD curves, using the following equation

(1) $$C\;\;=\;\;\frac{I}{[dE\mathrm{/d}t]\times s}\approx \frac{I}{[\Delta E/\Delta t]\times s}(\mathrm{mF}\;\;{\mathrm{cm}}^{-2})$$

where *I* is the constant discharge current, Δ*t* is the discharge time interval, *s* indicates the area of the corresponding active electrode material, and Δ*E* represents the voltage change after a full discharge.

A two‐electrode system configuration (V2O5/SWCNTs fiber positive electrode and VN/SWCNTs fiber negative electrode) was used to measure the performance of the FASCs devices in the PVA/KOH solid‐state gel electrolyte.

The energy density (*E*) of the FASCs device was deduced by Equation (2)

(2) $$E\;=\;0.5CV^{2}(\mu {\mathrm{Wh cm}}^{-2})$$

The power density (*P*) of the FASCs device was obtained by the energy density (*E*) and discharging time (*t*) according to the following equation

(3) $$P\;\;=\;\;\frac{E}{t}\;\;(\mu {\mathrm{W cm}}^{-2})$$

*The Integration of Flexible Wearable FASCs Device and Fiber‐Shaped Temperature (FT) Sensor*: The rGO fiber serves as temperature‐sensitive material of temperature sensor, which is wrapped on the FASCs device.

*The Integrated Configuration Measurement*: Electrical properties of the integrated device were measured using a Keithley analyzer (model 4200) equipped with a heating stage.

*The Humidity Response of FTSs*: Humidity‐controlled solutions (50 mL) of 96%, 54% H_2_SO_4_, 42% H_2_SO_4_, saturated NaCl, saturated KNO_3_ were put in closed chambers of 500 mL, which, respectively, provide different constant RH at 25 °C: about 5, 30, 58, 75, and 95% after 24 h. The resistance of FTSs was measured when the device was placed in the chamber with different RH.

## Conflict of Interest

The authors declare no conflict of interest.

## Supporting information

Supplementary
