# Supplementary material for: 3D Printing Fiber Electrodes for an All‐Fiber Integrated Electronic Device via Hybridization of an Asymmetric Supercapacitor and a Temperature Sensor
Source: Adv Sci (Weinh). 2018 Sep 25;5(11):1801114. doi: 10.1002/advs.201801114 (PMC6247048; doi:10.1002/advs.201801114)
Supplement: Supplementary file 1 — Supplementary [file ADVS-5-1801114-s001.pdf]

Copyright WILEY-VCH Verlag GmbH & Co. KGaA, 69469 Weinheim, Germany, 2018.

ADVANCED SCIENCE
Open Access

Supporting Information

for *Adv. Sci.,* DOI: 10.1002/advs.201801114

3D Printing Fiber Electrodes for an All-Fiber Integrated Electronic Device via Hybridization of an Asymmetric Supercapacitor and a Temperature Sensor

Jingxin Zhao, Yan Zhang, Yinan Huang, Jixun Xie, Xiaoxin Zhao, Chaowei Li, Jingyi Qu, Qichong Zhang, Juan Sun, Bing He, Qiulong Li, Conghua Lu, Xinhua Xu, Weibang Lu, Liqiang Li, and Yagang Yao**

3D printing fiber electrodes for all-fiber integrated electronic device via hybridization of an asymmetric supercapacitor and a temperature sensor

By Jingxin Zhao[+], Yan Zhang[+], Yinan Huang[+], Jixun Xie, Xiaoxin Zhao, Chaowei Li, Jingyi Qu, Qichong Zhang, Juan Sun, Bing He, Qiulong Li, Conghua Lu, Xinhua Xu, Weibang Lu, Liqiang Li, Yagang Yao**

Prof. C. H. Lu, Prof. X. H. Xu, Dr. J. X. Zhao, Dr. Y. Zhang, Dr. J. X. Xie, Dr. X. X. Zhao, Dr. J. Y. Qu

School of Materials Science and Engineering, Tianjin University, Tianjin, 300072, P. R. China

Prof. Y. G. Yao, Prof. L. Q. Li, Prof. W. B. Lu, Dr. J. X. Zhao, Dr. Y. N. Huang, Dr. C. W. Li, Dr. Q. C. Zhang, Dr. J. Suan, Dr. B. He, Dr. Q. L. Li

Division of Advanced Nanomaterials, Key Laboratory of Nanodevices and Applications, CAS Center for Excellence in Nanoscience, Suzhou Institute of Nano-tech and Nano-bionics, Chinese Academy of Sciences, Suzhou 215123, P. R. China

Prof. Y. G. Yao, Dr. J. X. Zhao, Dr. Y. N. Huang, Dr. C. W. Li, Dr. Q. C. Zhang, Dr. J. Suan, Dr. B. He, Dr. Q. L. Li

Division of Nanomaterials, Suzhou Institute of Nano-Tech and Nano-Bionics, Nanchang, Chinese Academy of Sciences, Nanchang 330200, China

E-mail: [*] ygyao2013@sinano.ac.cn

chlu@tju.edu.cn

[+] These authors contribute equally to this work.

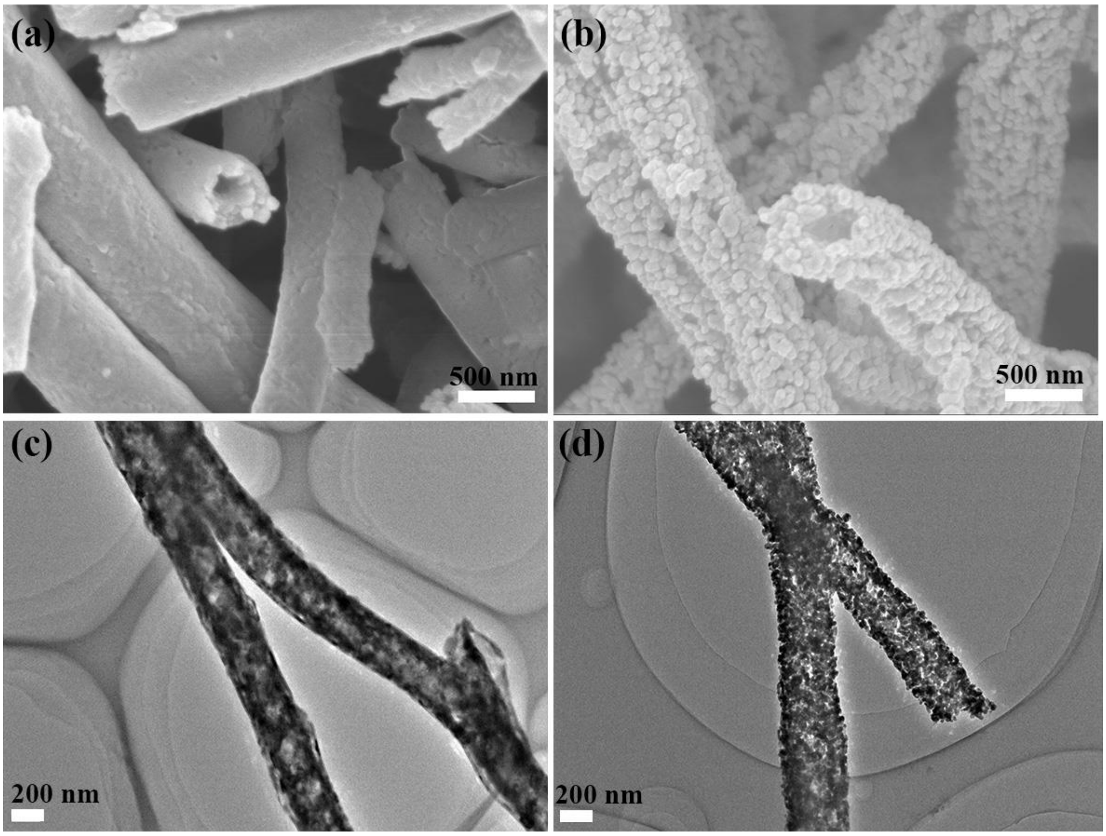

Figure S1. SEM images of (a) V_2O_5 and (b) VN samples, TEM images of (c) V_2O_5 and (d) VN samples.

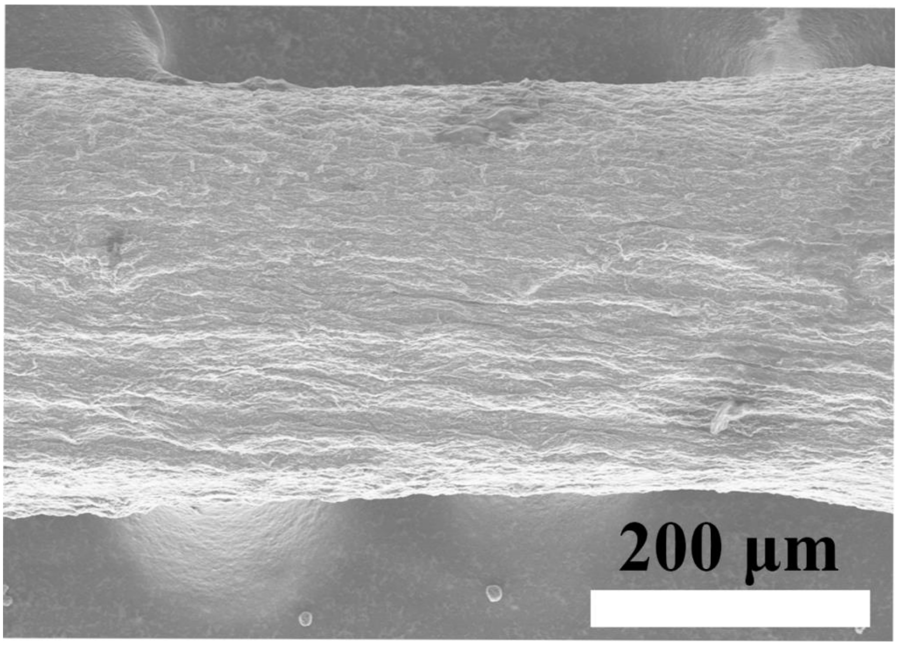

Figure S2. Low-magnification SEM image of the as-prepared VN/SWCNTs fber.

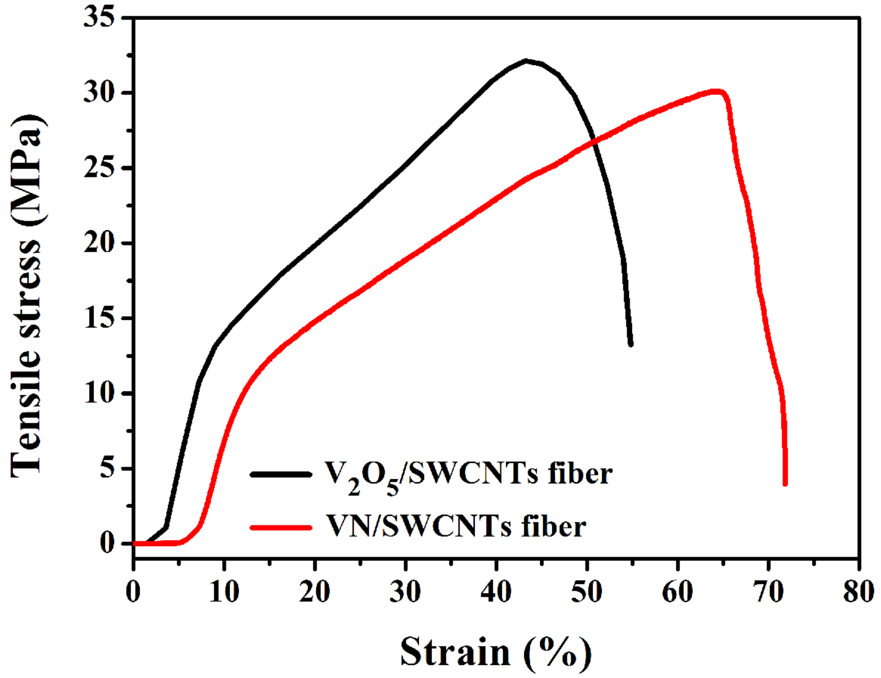

Figure S3. Stress-strain curves of the V_2O_5/SWCNTs and VN/SWCNTs fiber electrodes.

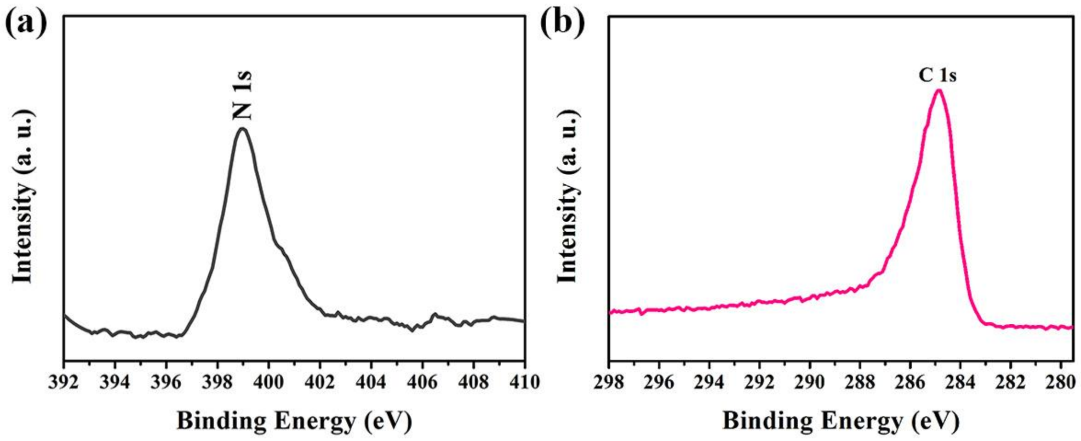

Figure S4. XPS spectra of as-prepared VN/SWCNTs fiber. (a) N 1s and (b) C 1s.

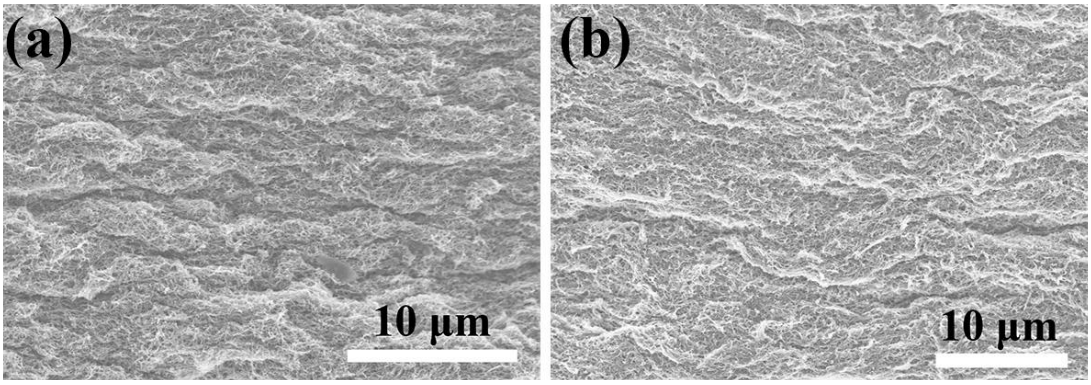

Figure S5. High-magnification SEM images of (a) V_2O_5/SWCNTs fiber and (b) VN/SWCNT fiber.

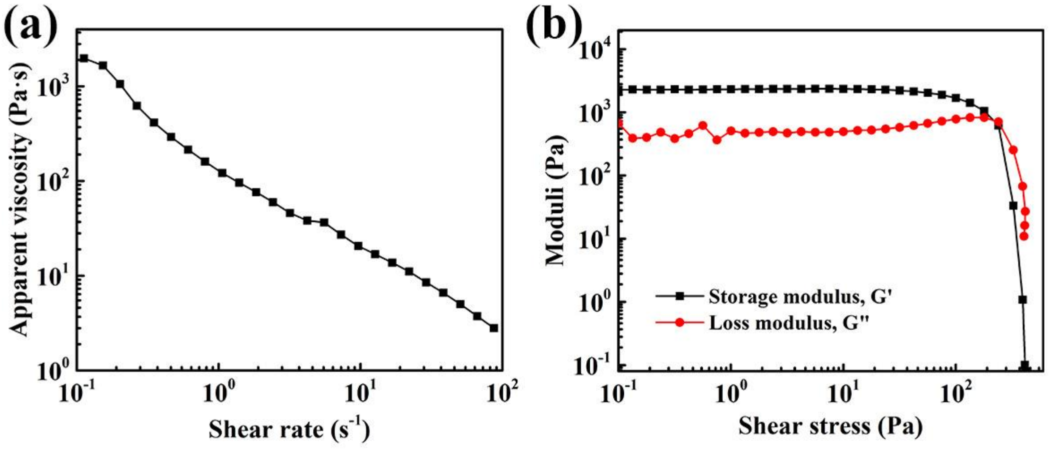

Figure S6. Rheological properties of pure SWCNTs slurry. (a) Apparent viscosity as a function of shear rate for pure SWCNTs slurry. (b) Storage modulus, G′, and loss modulus, G″, as a function of shear stress for pure SWCNTs slurry.

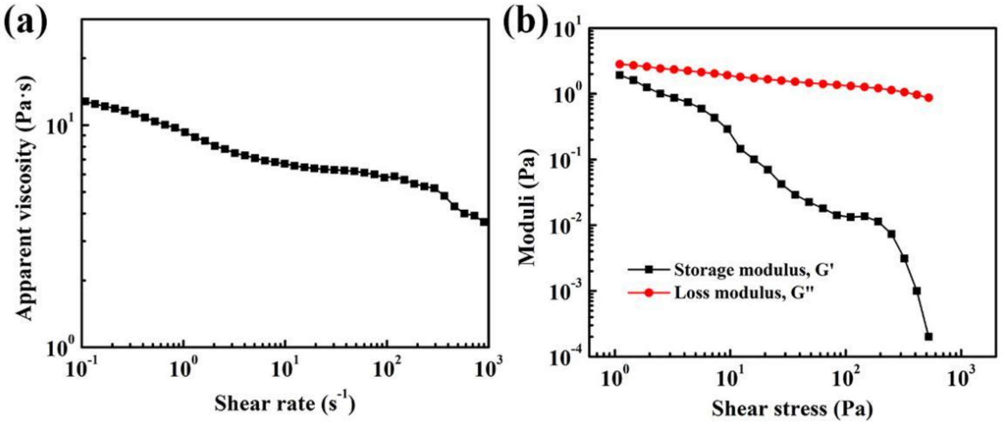

Figure S7. Rheological properties of electrolyte ink. (a) Apparent viscosity as a function of shear rate. (b) Storage modulus, G′, and loss modulus, G″, as a function of shear stress.

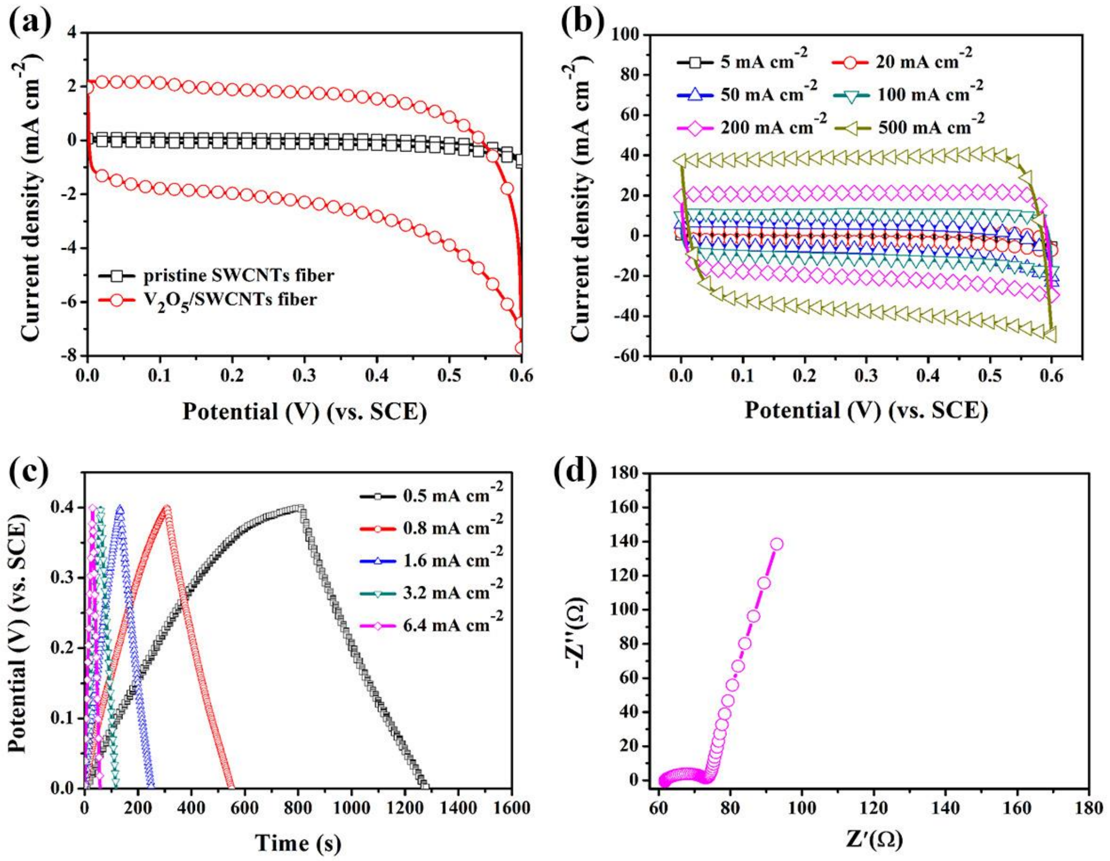

Figure S8. (a) CV of the V_2O_5/SWCNTs fiber in comparison with pure SWCNTs fiber. (b) CV curves at different scan rates. (c) GCD curves at different current densities. (d) Nyquist plot.

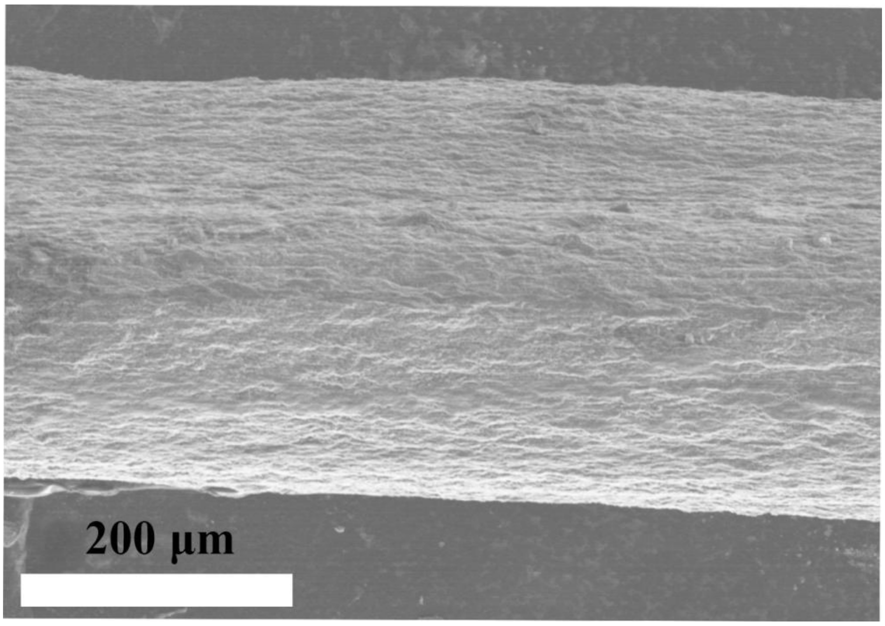

Figure S9. Low-magnification SEM image of pure SWCNTs fiber.

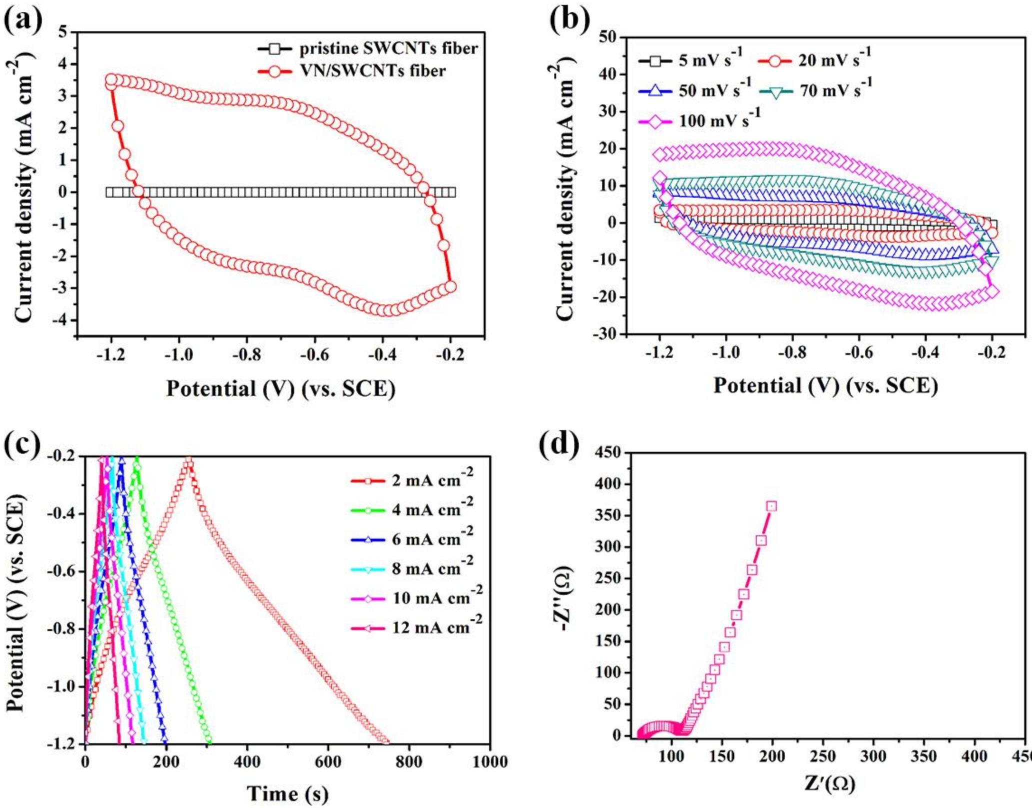

Figure S10. (a) CV of the VN/SWCNTs fiber in comparison with pristine SWCNTs fiber. (b) CV curves at different scan rates. (c) GCD curves at different current densities. (d) Nyquist plot.

Fig. S10(a) shows the CV of the VN/SWCNTs fiber electrode in comparison with the pristine SWCNTs fiber electrode determined at a scan rate of 25 mV s^{-1}. Comparing to the CV of the VN/SWCNTs fiber electrode, the signal from the pristine SWCNTs fiber electrode was negligible. The CV curves of VN/SWCNTs fiber negative electrode taken between -1.2 and -0.2 V in 1 M KOH electrolyte at different scan rates are shown in Fig. S10(b). Non-rectangular form of CV curves is due to the pseudocapacitive contribution of VN, which is attributed to redox reactions of the functional groups (C-O and O-C= O) on the surface of the VN.[1] GCD curves (Fig. S10(c)) reveal the relationship between potential (V) and time (t). The shape of the GCD curves shows the characteristic pseudocapacitance and the maximum specific capacitance is 983.28 mF cm^{-2} at a current density of 2 mA cm^{-2}, and the specific capacitance of 539.52 mF cm^{-2} is obatined at the current density of 12 mA cm^{-2}, indicating the good rate capability. The Nyquist plots (Fig. S10(d)) indicate a value of ~38.6 Ω for the equivalent series resistance (ESR) of the electrode.

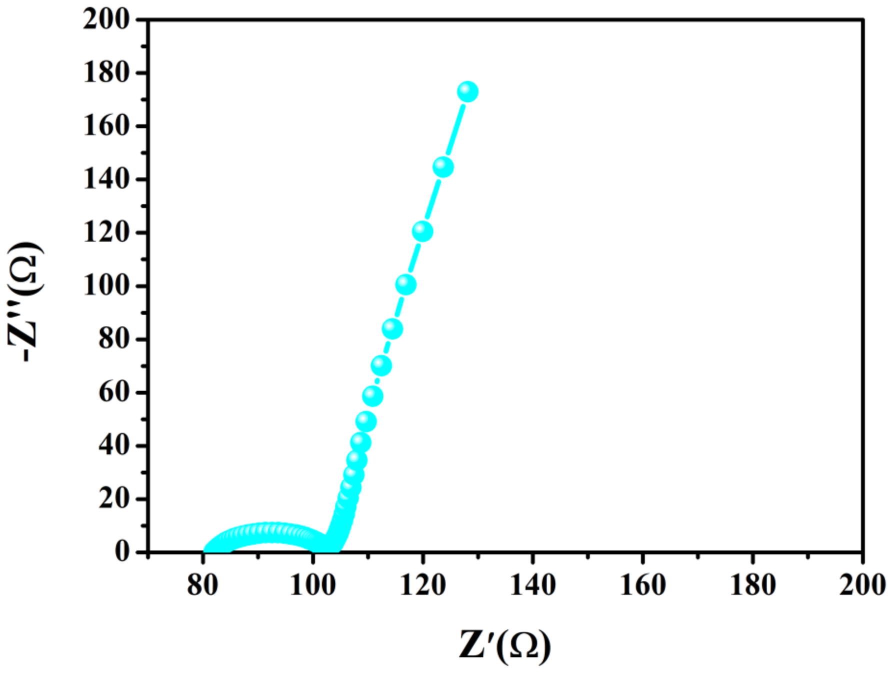

Figure S11. Nyquist plot of the assembled device.

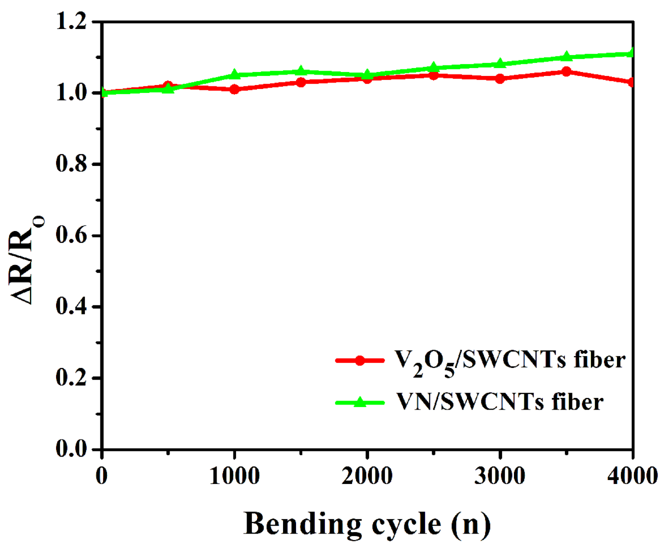

Figure S12. The electrical properties of V_2O_5/SWCNTs and VN/SWCNTs.

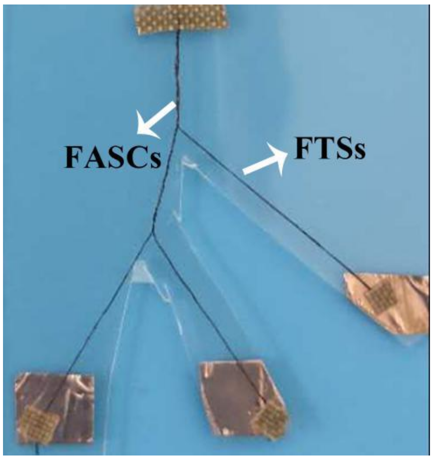

Figure S13. The photograph of FASCs/FTSs integrated configuration.

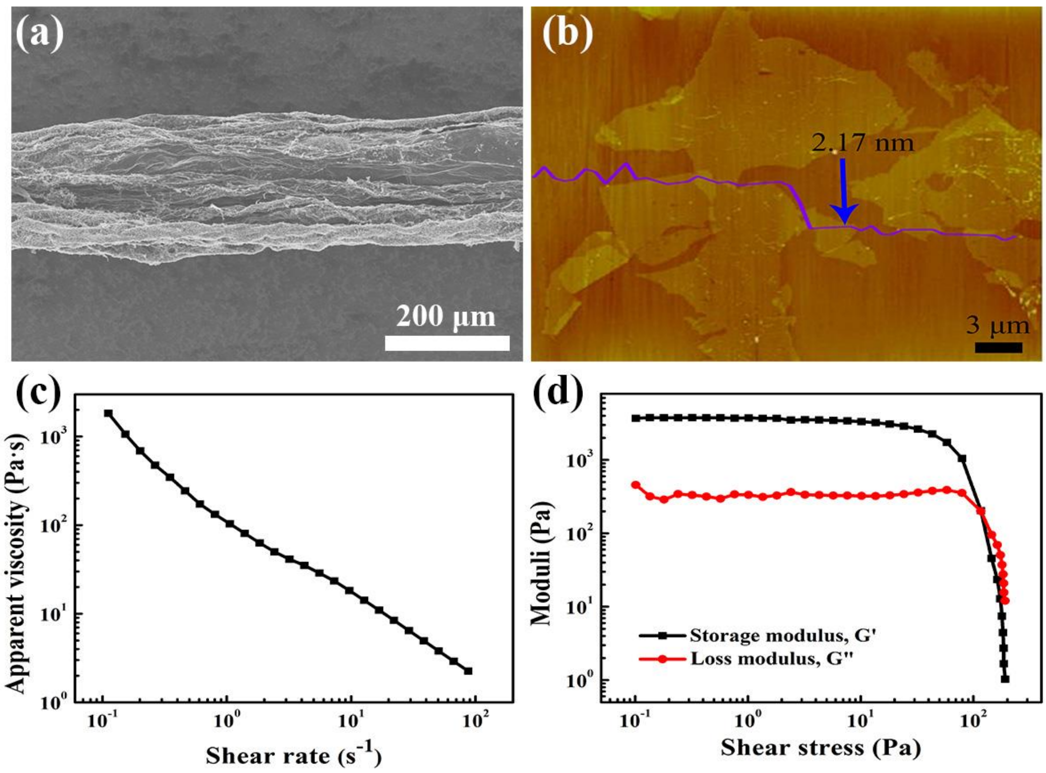

Figure S14. (a) SEM image of pure rGO fiber at low magnification. (b) Typical atomic force microscopy image of GO sheets. (c) Apparent viscosity as a function of shear rate for pure GO slurry. (d) Storage modulus, G′, and loss modulus, G″, as a function of shear stress for pure GO slurry.

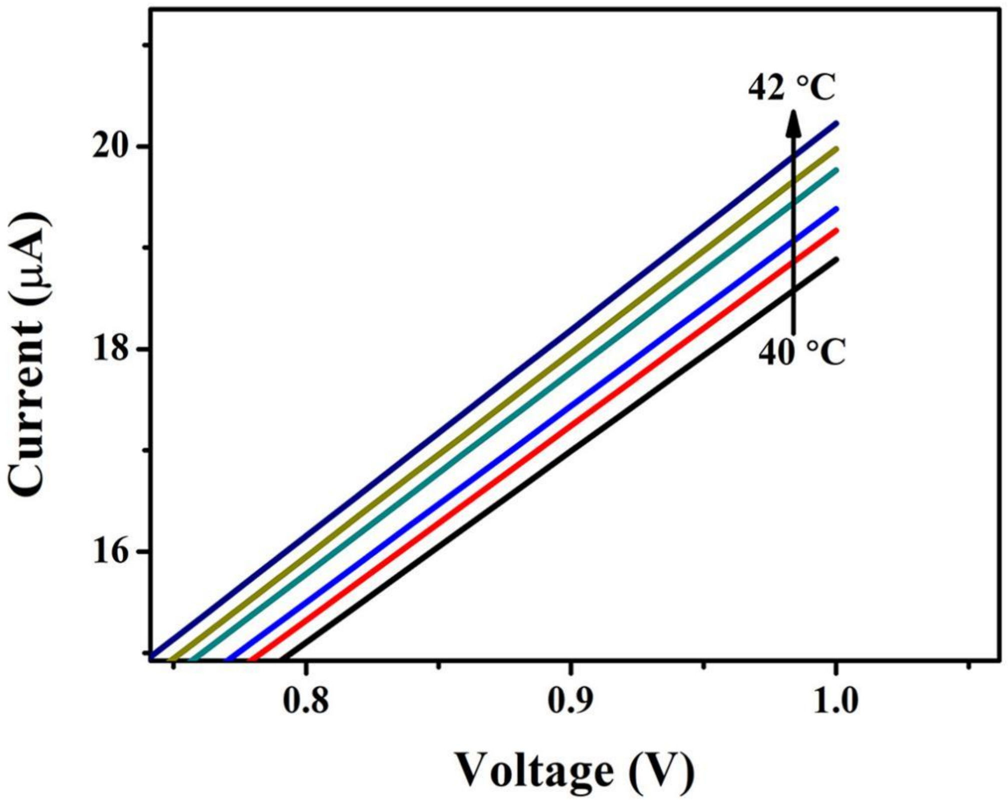

Figure S15. *I*-*V* curves of the integrated device in the temperature range of 40 to 42 ℃ (temperature step set as 0.4 ℃ for clarity).

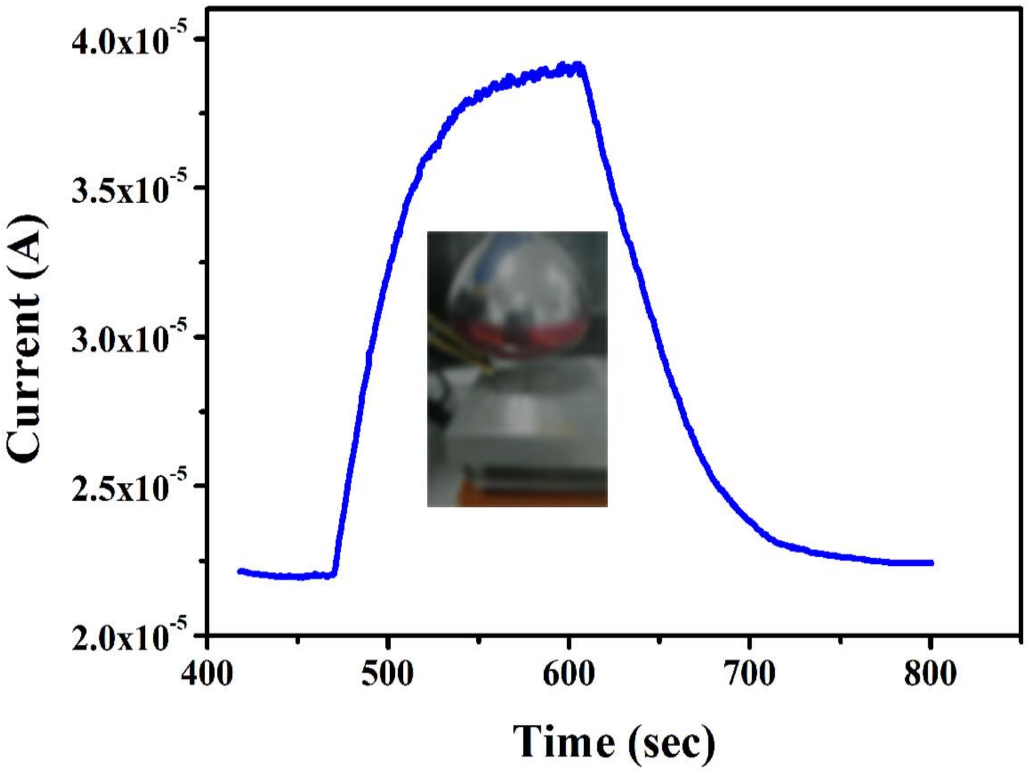

Figure S16. Time-dependent current measurements of the integrated device under infared radiation with an infrared light.

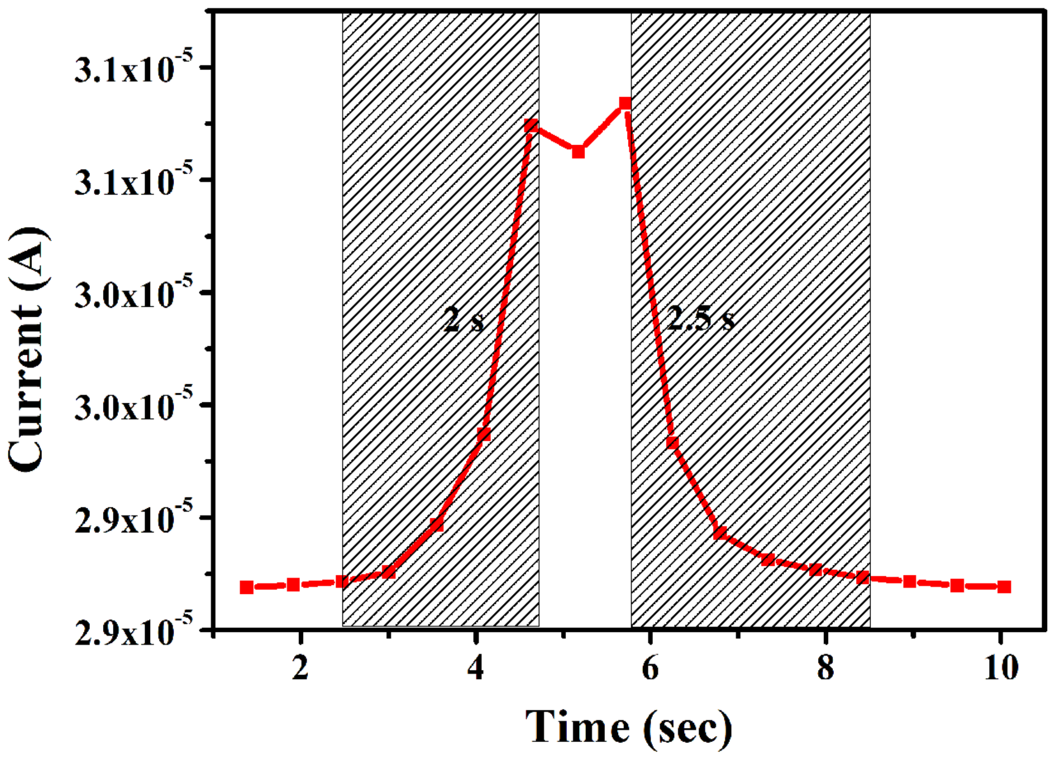

Figure S17. Rapid response/recovery time of integrated configuration.

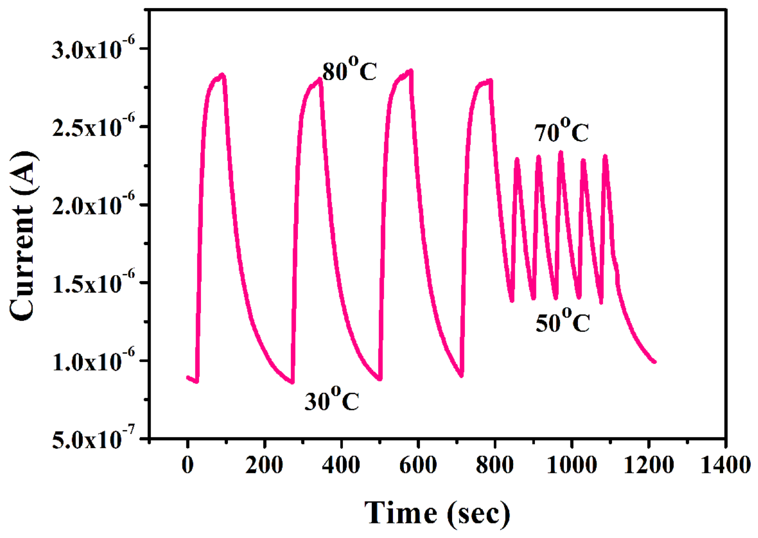

Figure S18. The repeatability of the integrated configuration.

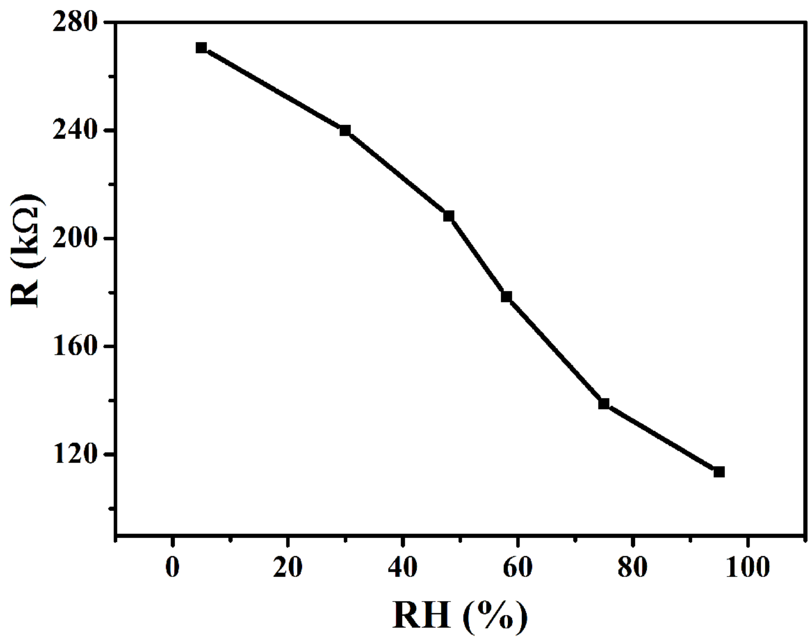

Figure S19. The humidity response of FTSs.

Reference

[1] J. X. Zhao, C. W. Li, Q. C. Zhang, J. Zhang, X. N. Wang, Z. Y. Lin, J. J. Wang, B. Lv, C. H. Lu, C. -P. Wong, Y. G. Yao, *J. Mater. Chem. A* **2017**, 5, 6928.
